# Nanoscale Metal-Organic Frameworks as Fluorescence Sensors for Food Safety

**DOI:** 10.3390/antibiotics10040358

**Published:** 2021-03-28

**Authors:** Xilin Dou, Kai Sun, Haobin Chen, Yifei Jiang, Li Wu, Jun Mei, Zhaoyang Ding, Jing Xie

**Affiliations:** 1 College of Food Science and Technology, Shanghai Ocean University, Shanghai 201306, China; a1douxilin@163.com (X.D.); jmei@shou.edu.cn (J.M.); 2 Department of Chemistry, University of Washington, Seattle, WA 98195, USA; sunkai@uw.edu (K.S.); chhb@uw.edu (H.C.); yifeij@uw.edu (Y.J.); 3 School of Public Health, Nantong University, Nantong 226019, China; wuli8686@ntu.edu.cn

**Keywords:** NMOFs, sensor, fluorescence, food safety

## Abstract

Food safety has attracted attention worldwide, and how to detect various kinds of hazardous substances in an efficient way has always been a focus. Metal-Organic Frameworks (MOFs) are a class of hybrid porous materials formed by organic ligand and metal ions. Nanoscale MOFs (NMOFs) exhibit great potential in serving as fluorescence sensors for food safety due to their superior properties including high accuracy, great stability, fast response, etc. In this review, we focus on the recent development of NMOFs sensing for food safety. Several typical methods of NMOFs synthesis are presented. NMOFs-based fluorescence sensors for contaminants and adulterants, such as antibiotics, food additives, ions and mycotoxin etc. are summarized, and the sensing mechanisms are also presented. We explore these challenges in detail and provide suggestions about how they may be surmounted. This review could help the exploration of NMOFs sensors in food related work.

## 1. Introduction

During the past few decades, food safety issues happened worldwide, such as dioxin in chicken feed, melamine in infant formula, and the outbreak of some foodborne pathogen [1]. As a consequence of these incidents, people are eager for safe food products. Food safety analysis plays a significant role in the food safety area to protect people from risk. However, effective analysis methods for food safety are always challenging because of food matrices possessing a very complicated structure and severe interference originating from a co-existing substance during the analysis process [2]. Facile and sensitive analysis methods for in situ and real-time sensing are urgently needed. Fluorescence-based analysis emerged as a promising method to improve safety sensing [3,4,5,6]. Quantum dots, organic fluorescence dyes, and metallic nanoclusters are classical fluorescent materials; however, these materials inevitably showed weakness such as toxicity, poor selectivity and stability, and high costs [7,8,9]. Sensitive, selective, and cheap fluorescence materials are urgently required to design fluorescence sensors.

MOFs are a class of hybrid porous materials formed by organic ligand and metal ions [10,11]. Owing to the abundant combination of metals and organic ligands, high surface areas, and numerous adjustable pores, MOFs have been applied for a variety of fields [12], such as energy storage [13], gas storage [14], catalysis [15], biomedical imaging and drug delivery [16], separation [17], adsorption [18], sensing [19,20,21,22,23], etc. However, bulk-size MOFs for use as fluorescence sensors suffer from some shortcomings like slow signal response, poor dispersity as well as stability and that application is difficult in the liquid phase [24,25]. In order to overcome these problems, many research groups have scaled down to the nano size to prepare NMOFs using as fluorescence sensors. NMOFs-based fluorescence sensors were applied more and more in food analysis because of their outstanding properties for fluorescence detection and delicate structure that can be tailored. In addition, these trials have showed that NMOFs have exciting potential in the food safety area. 

In this review, the following overviews will be covered: Firstly, the general synthetic methods for NMOFs are induced by some typical instances. Then, an overview of the applications of NMOFs as fluorescence sensors applying for the detection of risky small molecules, ions, and food-borne pathogens in food is provided, and the sensing mechanisms are interpreted. Finally, future perspectives and challenges of NMOFs as fluorescence sensors in the field of food safety are discussed.

## 2. Synthesis of NMOFs

Solvothermal methods, microwave, and sonochemical approaches, microemulsion or reverse microemulsion syntheses and surfactant-mediated or templated solvothermal/hydrothermal methods are general synthetic methods for NMOFs [26]. 

**Figure 1:**
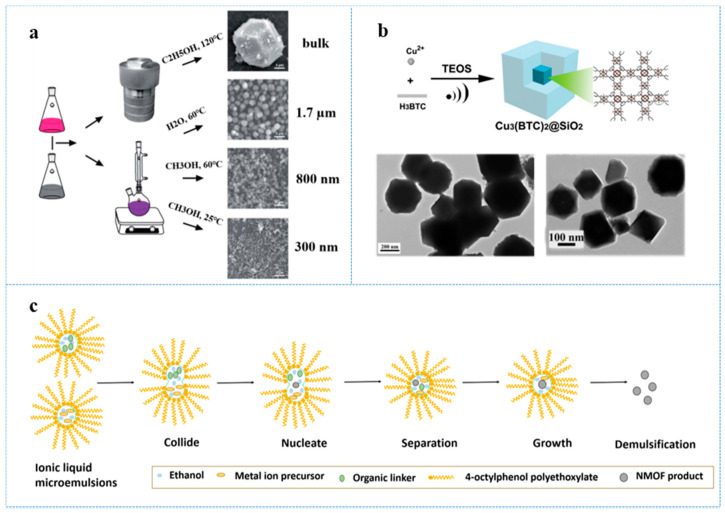
(**a**) Schematic illustration of synthesis of ZIF-67 with various sizes by solvothermal methods. Scale bars = 3 μm; reprinted with modifications from Xia et al. [28]; (**b**) schematic representation of the synthesis of the Cu_3_(BTC)_2_@SiO_2_ core–shell nanocrystals using ultrasonic method and the TEM images of as-synthesized Cu_3_(BTC)_2_@SiO_2_; reprinted with modifications from Li et al. [32]; (**c**) schematic illustration of the growth mechanism of HKUST-1 nanoparticles prepared in an ionic liquid-containing microemulsion system, adapted from Zheng et al. [33].

Solvothermal methods are the most extensive and effective methods for the synthesis of MOFs in all sizes. During the utility of solvothermal methods for NMOF synthesis, controlling reaction conditions and selecting chemical modulator can help us to get MOFs for a specific size [27]. For example, by methods of changing reaction temperature, time, and solvent, Xia et al. [28] synthesized ZIF-67 crystals of varying sizes. Bulk ZIF-67 was synthesized in ethanol at 120 °C for three days. In addition, 1.7 μm crystals were prepared from deionized water at 60 °C for 20 h while 800 nm crystals were obtained from methanol at 60 °C for 20 h. In addition, the smallest crystals with particle sizes of 300 nm were also synthesized in methanol but at 25 °C for 20 h (Figure 1a). Introducing various concentrations of modulators also leads to different crystallite sizes via the competition of metal binding sites and decrease of available nucleation sites. Modulated solvothermal MOF preparation was presented by Schaate et al. [29], where the sizes and morphology of prepared Zr-based MOFs were controlled through adding different amounts of benzoic acid. 

Microwave heating accomplishes rapid synthesis and enhanced kinetics of crystal nucleation via rapid and efficient energy transfer as well as high instantaneous temperature, which makes for MOF size reduction. Bunzen et al. [30] found that, by methods of microwave-assisted synthesis, uniform benzenetriazolate Zn-based MOFs—MFU-4 cubic nanocrystals (104 ± 4 nm) can be obtained with a pretty high reaction yield in a DMF−EtOH (1:1) mixture at 100 °C for just 2 min, the reaction time was much shorter than solvothermal synthesis. Sonochemical approaches for NMOFs preparation also allow rapid kinetics and crystals morphology and phase controlling. The mechanism is that the growth and collapse of acoustic cavitations make high temperatures (>5000 K) and pressures [31]. Li et al. [32] synthesized uniform core–shell nanoparticles (20–400 nm) of Cu_3_(BTC)_2_@SiO_2_ by sonochemical approaches. These nanoparticles are accessed by mixing both the NMOF and SiO_2_ shell precursors in an ultrasonic solution. In addition, the MOF nanocrystals formed in a short time, followed by growth of SiO_2_ on the surface of the NMOFs (Figure 1b).

Through microemulsion or reverse microemulsion syntheses, the size and structure of MOFs can be easily controlled. ZIF-8 and ZIF-67 nanoparticles were prepared in H_2_O/1-butyl-3-methylimidazolium hexauorophosphate/4-octylphenol polyethoxylate microemulsion through direct mixing by Zheng et al. [33]. Adding ethanol to the ionic liquid-containing microemulsion system, HKUST-1, an MOF with benzene-1,3,5-tricarboxylate as an organic ligand formed. The coordination reaction of MOFs happened in water droplets (Figure 1c). The average particle sizes reached 2.2 nm, 2.3 nm, and 1.6 nm for ZIF-8, ZIF-67, and HKUST-1, respectively. Seoane et al. [34] achieved hierarchical MOF nanocrystals’ synthesis through the surfactant-mediated method.

In addition, some unconventional methods were also exploited for NMOF synthesis, such as centrifugation [35], self-exfoliation [36] and spray-drying [37].

## 3. NMOFs for Food Quality

### 3.1. Small Molecules

#### 3.1.1. Antibiotics

Antibiotics, one of the veterinary drugs, have been extensively used for preventing and treating diseases in animal growth, while the abuse of antibiotics might cause severe antibiotic residues in animal food and water [38], which may pose potential negative effects to consumers.

**Figure 2:**
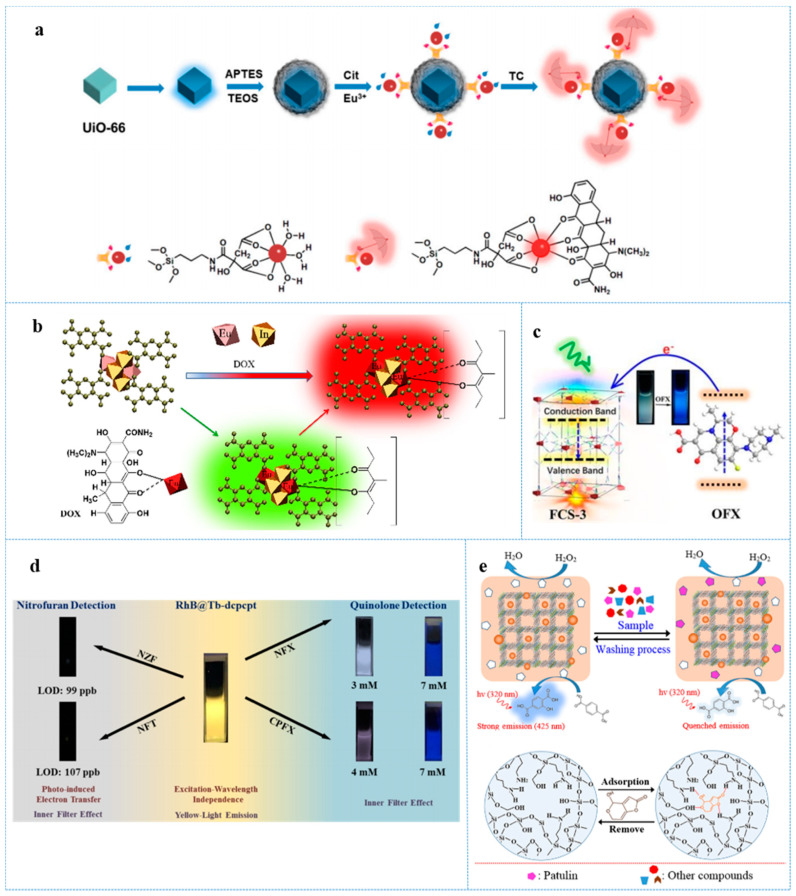
(**a**) Schematic diagram for the fabrication procedure of a Dye@UiO-66-@SiO_2_-Cit-Eu nano-probe and the sensing process for tetracycline (TC). Reprinted with modifications from Jia et al. [39]; (**b**) schematic illustration showing the recognition of DOX based on the fluorescent sensor Eu-In-BTEC, reprinted with modifications from Yu et al. [43]; (**c**) fabrication and engineering of anionic metal–organic framework as a unique turn-on fluorescent chemical sensor for ultra-sensitive detection of antibiotics, reprinted with modifications from Li et al. [44]; (**d**) The image of RhB@Tb-dcpcpt for sensitive and selective detection toward antibiotics in water, reprinted with modifications from Yu et al. [48]; (**e**) Schematic image for selective determination of patulin based on MIP-capped AgNPs@ZnMOF and closer look on the location of patulin in MIP sites and its interaction with specific functional groups. Reprinted with modifications from Nafiseh Bagheri et al. [50].

Recently, a UiO-66-based dual-emission fluorescence probe (Dye@UiO-66-@SiO_2_-Cit-Eu) was presented for ratiometric determination of tetracycline (TC) [39]. In this case, fluorescence brightener (dye) was doped in UiO-66 to secure enough dispersity of the dye molecules. In addition, then, the dye-doped UiO-66 was encapsulated in a silica shell to not only prevent the dye from loss, but also facilitate grafting with Eu^3+^. This fluorescence probe exhibited a blue fluorescence emission at 430 nm derived from the dye, which served as the reference emission. In the presence TC, this probe emitted a strong red fluorescence at 617 nm (response emission) that was derived from Eu^3+^. Meanwhile, the reference emission remained unchanged (Figure 2a). Through using the I_617_/I_430_ ratio as a ratiomatric signal output, the NMOF-based fluorescence probe provided a selective and sensitive determination of TC with an LOD of 17.9 nM. Interestingly, based on the above mechanism, a practical and facile paper-based probe was developed by methods of fixing Dye@UiO-66@SiO2-Cit-Eu on filter paper for effective detection of TC. Under excitation wavelength of 365 nm, the color of the paper would change from blue to red as the concentration of TC increasing. This paper-based probe realized a real-time, cost-effective and rapid sensing for TC in situ with an LOD of about 0.1 µM. 

An indium-based NMOF (In-sbdc) was prepared with trans-4,4′-stilbenedicarboxylate as an organic ligand for sensitive detection of tetracycline type antibiotics (tetracycline, chlorotetracycline, and oxytetracycline) [40]. In-sbdc can emit robust fluorescence under the excitation of 327 nm, which originated from the ligand. Upon the addition of TC antibiotics, the fluorescence emission was outstandingly quenched through the fluorescence resonance energy transfer (FRET) within five seconds. Meanwhile, In-sbdc possessed excellent stability, reusability, and selectivity with Na_2_S_2_O_4_ as the masking agent. However, in the food samples such as milk, fish and pork, In-sbdc can maintain a great sensing ability. The LODs of In-sbdc for tetracycline, chlorotetracycline, and oxytetracycline were 0.28 µM, 0.30 µM, and 0.30 µM, respectively.

Li et al. [41] reported a novel nanoscale hierarchical dual-metal organic framework (Al-MOF@Mo/Zn-MOF), which can ultra-sensitively detect TC antibiotics including doxycycline (DOX), tetracycline (TET), oxytetracycline (OTC), and chlortetracycline (CTC). In the presence of TC antibiotics, Al-MOF@Mo/Zn-MOF can be effectively quenched via the inner-filter effect (IFE) and the photo-induced electron transfer (PET) from this fluorescence probe to TC antibiotics. Al-MOF@Mo/Zn-MOF showed special 3D flower-like hierarchical nanostructure, which endowed this fluorescence probe with a large surface area, great porosity, and more active sites to recognize and absorb TCs, much improved mass transfer between Al-MOF@Mo/Zn-MOF and TCs and alleviated diffusion barrier from the solid–liquid interface. In addition, the metal nodes (Al, Zn and Mo) of this probe can effectively chelate with TCs to enhance the recognition ability, in particular, Mo, as a transition metal, could act as a Lewis basic to effectively recognize TCs. Both the special hierarchical structure and metal nodes of this probe enhanced the pre-concentration effect, in which part of the analytes are firstly absorbed into the MOFs, making the analytes’ contact with MOFs more sufficient [42], which endowed Al-MOF@Mo/Zn-MOF with excellent sensitivity with LODs of 0.56, 0.53, 0.58, and 0.86 nM for DOX, TET, OTC, and CTC, respectively. 

Nanoscale In-codoped Europium-based MOFs (Eu-In-BTEC) were prepared by introducing In into Eu-BTEC without further modification, which realized highly selective and sensitive turn-on detection of DOX in fish and urine [43]. Eu-In-BTEC exhibited non-emission in aqueous solution. Upon the addition of DOX, Eu-In-BTEC presented special dual channel performance—fluorescence enhanced at both 526 nm and 617 nm, which were originated from Eu and DOX itself, respectively. Then, the emission intensity at 526 nm gradually reduced while the intensity at 617 nm continuously increased and reached the maximum after 12 min (Figure 2b). The mechanism of detection is that the Eu-In-BTEC-DOX complex was generated during the sensing process, and the FRET between Eu-In-BTEC and DOX enabled the Eu to have luminescence. The LOD of Eu-In-BTEC for DOX reached as low as 47 nM. Surprisingly, Eu-In-BTEC was able to accurately detect DOX from other TCs possessing a similar structure through emitting fluorescence of special emission wavelength. The superior selectivity can be attributed to the pretty robust combination between DOX and Eu-In-BTEC. 

Anionic Zinc-based MOF sensors (FCS-3) achieved a rare turn-on emission determination of fluoroquinolone antibiotics including ofloxacin (OFX) with 5-[N,N-bis(5-methylisophthalic acid)amion] isophthalic acid (H_6_bmipia) as carboxylate ligand [44]. In the presence of fluoroquinolone antibiotics, the fluorescence emission of FCS-3 that originated from H_6_bmipia was dramatically enhanced while the addition of other antibiotics would result in the emission quenching of FCS-3. The mechanism of emission enhancement might be the strong interaction between FCS-3 and fluoroquinolone antibiotics as well as the PET from fluoroquinolone antibiotics to FCS-3 (Figure 2c). The LOD of FCS-3 for OFX reached up to 0.52 μM in aqueous solution. 

Li et al. [45] recently developed a polynuclear MOF (Tb-L1) with 1D channels for ratiometric determination of TC in ethanol. Tb-L1 showed both powerful representative emission of Tb^3+^ at 543 nm and the weak emission from organic ligand H_3_L1 at 345 nm. Upon addition of TC, the fluorescence emission of Tb^3+^ was extremely quenched while the emission of the ligand accordingly enhanced. The sensing mechanism is that the IFE efficiently impaired the excitation for the MOF, which impacted the energy transfer from the ligand to Tb^3+^. After calculating the I_543_/T_345_ ratio, Tb-L1 revealed outstanding sensitivity for TC with an LOD of 8 ng/mL using a ratiometric response.

Zhu et al. [46] designed and prepared sensitive Cadmium-based MOFs with six-node dendritic 1,3,5-tris [3,5-bis(3-carboxylphenyl-1-yl)phenyl-1-yl]benzene as organic ligands. This sensor was utilized for selective determination of nitrofurazone (NFZ) and nitrofurantoin (NFT) that are widely used as food supplements in animal husbandry and aquaculture industry. The sensing mechanism is that NFZ or NFT can quench fluorescence emission of the sensor a lot through energy competition with quenching efficiencies of 81% and 87%, respectively. Meanwhile, this sensor possesses great selectivity towards NFZ or NFT and outstanding reusability.

Two fluorescence sensors were prepared based on ZIF-8 and RhB and fluorescein disodium salt (FSS), for sensitive detection of nitrofurans (NFAs) and TCs in the water medium [47]. In the presence of NFAs, the dramatical fluorescence quenching was observed while, upon the addition of TCs, the fluorescence enhancement was observed. The quenching efficiencies can be ascribed to the combination of the PET and the FRET. Meanwhile, the mechanism of fluorescence enhancement for TCs might be that TC molecules restricted conformational rotation of ZIF-8. The LOD of RhB@ZIF-8 for nitrofurantoin NFT, NFZ, TC, and OTC were 0.26 µM, 0.47 µM, 0.11 µM, and 0.14 µM, respectively. In addition, FSS@ZIF-8 for NFT, NFZ, TC, and OTC were 0.31 µM, 0.35 µM, 0.17 µM, and 0.16 µM.

RhB@Tb-dcpcpt were fabricated by incorporating RhB into the channels of MOFs, [Me_2_NH_2_][Tb_3_(dcpcpt)_3_(HCOO)]∙DMF∙15H_2_O (Tb-dcpcpt), which accomplished sensitive detection of nitrofuran antibiotics (NFZ and NFT) and quinolone antibiotics including ciprofloxacin (CPFX) and norfloxacin (NFX) in water medium [48]. RhB@Tb-dcpcpt presented rare excitation-wavelength-independent fluorescence in aqueous solution. In the whole range of 300–390 nm (excitation), the RhB@Tb-dcpcpt remained a stable yellow fluorescence emission. In the presence of NFZ and NFT, the sensor was obviously quenched. Interestingly, this sensor can selectively detect quinolone antibiotics via the luminescence-color-changing process. Upon the addition of CPFX or NFX, the fluorescence emission of RhB@Tb-dcpcpt remarkably changed from yellow to blue, while other antibiotics cannot cause the process (Figure 2d). The mechanism of sensing may be ascribed to the PET and IFE. The LOF of RhB@Tb-dcpcpt for NZF, NFT, CPFX, and NFX are as low as 0.502 µm, 0.448 µM, 0.21 µM, and 0.17 µM, respectively.

Cadmium-based MOFs (Cd-MOF) with 1,4-bis(2-methyl-imidazol-1-yl)butane and 1,2-phenylenediacetic acid (H2L) as organic ligand accomplished selective determination of ceftriaxone sodium (CRO), a common antibiotic [49]. The Cd-MOF showed robust emission in water that is derived from the ligand H2L. A remarkable and rapid (within 20 s) quenching effect with quenching efficiency of more than 90% can be observed when CRO was added, while, upon the addition of other antibiotics, the quenching efficiency was below 15%. The mechanism of quenching effect is possibly ascribed to the FRET between the Cd-MOF and the antibiotics. Meanwhile, the Cd-MOF exhibited great PH-independent characteristics. The result of detection was not affected when PH changed among PH = 4–11. The LOD for CRO reached 55 ppb.

Stable 2D Zinc-based MOF sensors with 1,2-benzenediacetic acid and 1, 1′-(1, 4-butanediyl) bis (imidazole) as organic ligand can detect not only antibiotics including chloramphenicol (CHL), CRO but also Ascorbic acid (AA) [50]. This sensor possessed excellent chemical stability in a wide PH range. In addition, CHL, CRO, or AA can effectively quench the fluorescence emission of this sensor within five seconds via energy absorption competition. The LOD of this sensor for CHL, CRO, and AA were as low as 12 ppb, 3.9 ppb, and 1.6 ppb, respectively.

A molecularly imprinted polymer (MIP)-capped AgNPs@ZnMOF was presented for serving as a sensitive sensor of patulin [51]. This sensor was composed of MIP that can adsorb patulin with excellent specificity and selectivity, Ag nanoparticles that possessed peroxidase-like activity, and NMOF that was used as support materials for MIPs as well as endowing this sensing platform with better catalytic activity than pure AgNPs due to its high surface area. An MIP-capped AgNPs@ZnMOF can catalyze terephthalic acid (TA)-H_2_O_2_ reaction to produce 2-hydroxy terephthalic acid, which can emit strong fluorescence emission. In the presence of patulin, the catalytic activity of this sensing platform was restrained, leading to obvious fluorescence quenching (Figure 2e). The LOD for patulin was as low as 0.06 µM.

#### 3.1.2. Food Additives

Food additives play an essential role in maintaining or enhancing safety, nutritional value, appearance, texture, freshness, and other characteristics of food. However, the abuse of food additives would result in a severe threat to both humans and animals [52], which cause concerns about food safety from people. Therefore, it is urgent to develop sensitive, rapid, and simple detection methods for various food additives. NMOF-based sensors possess the above properties and are able to accomplish accurate determination in the complicated real food samples through selecting and designing appropriate NMOFs.

**Figure 3:**
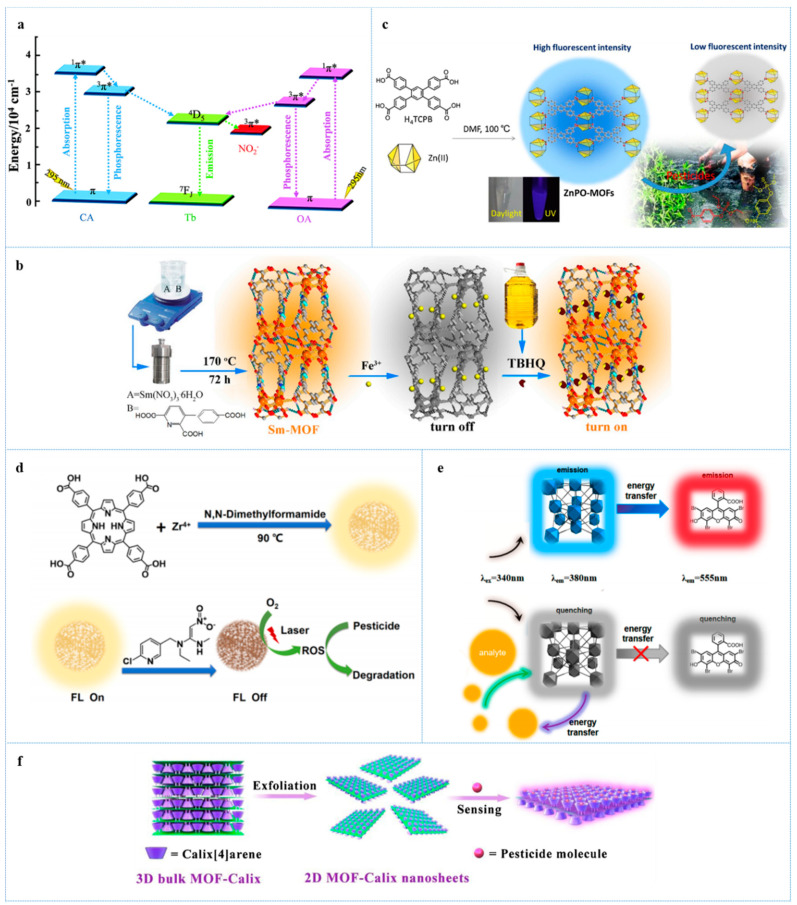
(**a**) the schematic energy transfer process of Tb-MOF in the presence of nitrite, reprinted with modifications from Min et al. [54]; (**b**) the detection mechanism of the “turn off-on” sensor (Sm-MOF) for TBHQ, reprinted with modifications from Liu et al. [59]; (**c**) Schematic diagram for synthesis of ZnPO-MOFs and their application for rapidly detecting organophosphorus pesticides, reprinted with modifications from Xu et al. [64]; (**d**) schematic illustration for the synthesis of FMOF and its fluorescent sensing and photocatalytic applications for NIT residue, reprinted with modifications from Liu et al. [66]; (**e**) schematic illustration showing the mechanism of EY@DUT-52 as a fluorescence sensor for pesticides, reprinted with modifications from Wei et al. [71]; (**f**) schematic illustration for the fabrication of 2D MOF-Calix nanosheets and sensitive detection of pesticide, reprinted with modifications from Yu et al. [72].

Nitrite is extensively used as preservatives in pickled products, but it can generate N-nitrosamines that is carcinogenic during digestion in the human body [53]. Min et al. [54] developed a Terbium-based MOF (Tb-MOF) with chelidonic acid (H_2_CA) and oxalic acid (H_2_OA) as an organic ligand, for fluorescence sensing nitrite in aqueous solution. Under the UV-light, the antenna effect happened in this fluorescence sensor—energy transfer from organic ligand (CA^2−^ and OA^2−^) to metal node Tb^3+^ leading to a bright green fluorescence emission. In the presence of nitrite, obvious quenching behavior within a short time was observed, which might be attributed to the energy transfer from this fluorescence sensor to nitrite. Meanwhile, this sensor can be reused more than five times and selectively detect for nitrite from kinds of salt (Figure 3a). The LOD for nitrite was as low as 28.25 nM.

Formaldehyde is widely applied as an industrial preservative and poses a potential risk to public health. However, some illegal vendors use formaldehyde as food preservatives to extend the shelf life of aquatic products [55]. Kowsalya Vellingiri et al. [56] found that a Zirconium-based NMOF (UiO-66-NH_2_) can serve as fluorescence sensor for selective determination of formaldehyde. Upon the addition of formaldehyde, the emission of UiO-66-NH_2_ was dramatically quenched due to the formation of a non-covalent bond between the C_6_H_3_NH_2_ unit in the NMOF and formaldehyde with an LOD of 4 ppm. A UiO-66-NH_2_-based sensor was also applied by Li et al. [57] for the detection of formaldehyde in aqueous solution. They developed dual-emissive heterometal-organic frameworks (Eu/Zr-MOFs) by doping Eu^3+^ into UiO-66-NH_2_. The emission from the organic ligand (2-aminoterephthalic acid) of Eu/Zr-MOFs served as a response signal and the characteristic emission of Eu^3+^ served as the reference signal. In the presence of formaldehyde, the fluorescence of 2-aminoterephthalic acid at 465 nm was greatly enhanced, which might be attributed to the electron transfer from amino groups of the ligand to formaldehyde. Meanwhile, the fluorescence of Eu^3+^ at 615 nm was negligibly improved. Through using the I_456_/I_615_ as a ratiometric readout, the LOD for formaldehyde was as low as 0.2 ppm. 

Tertiary butylhydroquinone (TBHQ) was widely used as antioxidants in edible oils and oil-containing foods due to its high antioxidant capacity and low price. Excessive intake of TBHQ will put a heavy burden on the liver [58]. A novel Samarium-based MOF (Sm-MOF) was designed with 3-(2,4-dicarboxylate phenyl)-2-pyridinecarboxylic acid as an organic ligand for turn-off-on detection of TBHQ in edible oil [59]. Sm-MOF exhibited bright orange fluorescence emission that can be almost completely quenched by Fe^3+^ in certain concentration. The quenching effect may be subjected to the binding interaction between Fe^3+^ and Sm-MOF. As shown in Figure 3b, in the presence of TBHQ, the quenched orange fluorescence emission recovered because TBHQ has a higher affinity with Fe^3+^ and thus destroyed the bond between Fe^3+^ and Sm-MOF. Owing to the space effect, electron effect, sensing process, and so on, Sm-MOF possessed superior selectivity to TBHQ with an LOD of 5.6 ng/mL.

Sesamol (3,4-methylenedioxyphenol) is a nature antioxidant and exhibited potential neuroprotective effects [60]. Wang et al. [61] reported a 3D alkaline NMOF (Tb@Sr-MOF) for selectively sensing sesamol. Tb@Sr-MOF was prepared by encapsulating Tb^3+^ into Strontium-based MOFs—[Sr(BDC)·DMAC·H_2_O]_n_ (BDC = benzene-1,4-dicarboxylate; DMAC = N,N-dimethylacetamide) and showed robust green fluorescence at 545 nm under the UV-light originated from Tb^3+^. In the presence of sesamol, the fluorescence emission of Tb@Sr-MOF was almost completely quenched. Meanwhile, the fluorescence emission at 330 nm, which was derived from sesamol itself, was enhanced. The quenching effect at 545 nm might be ascribed to the energy competition between sesamol and Tb@Sr-MOF. With I_343_/I_545_ as output, the LOD for sesamol was 4.2 µM.

#### 3.1.3. Pesticides

Pesticides play an essential role in the agricultural field to secure the quantity and quality of products [62]. However, many pesticides are hard to degrade in the human body and may lead to different kinds of diseases and even cancer. Thus, effective detection of pesticides makes a lot of sense. Using NMOF as fluorescence sensor for on-site sensing pesticides can overcome practical limitations of conventional methods like chromatographic techniques, which require professional operators, instruments, and so on [63]. Many research groups have applied NMOF sensors to effectively determine pesticides in water medium and real food samples. To date, the usual sensing mechanism of NMOFs-based fluorescence sensors for organic pesticides determination is the pesticides-induced quenching effect. Meanwhile, novel detecting methods and mechanisms are explored.

Xu et al. [64] found that a nanoscale Zinc-based MOF (ZnPO-MOF) with 1,2,4,5-Tetrakis(4-carboxyphenyl) benzene as an organic ligand can be quenched by parathion-methyl, a kind of organophosphate pesticides (OPs), and thus presented a rapid and selective parathion-methyl sensing with an LOD of 0.456 nM. The sensing mechanism is that the −NO_2_, an electron-withdrawing group, on the parathion-methyl giving rise to the PET from ZnPO-MOF to the target (Figure 3c).

Several NMOF-based fluorescence sensors reported by different research groups shared a similar mechanism. For example, two dye@MOFs fluorescence sensors (Rho B@1 and Rho 6G@1) were prepared by encapsulated luminescent molecules, Rhodamine B and Rhodamine 6G, into the channels of MOFs—[Cd_2_(tib)(btb)(H_2_O)_2_]∙NO_3_∙2DMF for ratiometric detecting pesticides [65]. In the presence of tested pesticides, the quenching effect with a different degree was observed, and the pesticides with stronger electron-withdrawing groups exhibited a greater quenching effect. When nitenpyram, a pesticide with strong electron affinity was added, and the fluorescence emission originated from the luminescent guest molecules slightly decreased, but the emission from the host MOFs was very quenched in a short time because of the PET from the MOFs to electron- withdrawing pesticides. The LOD of Rho B@1 and Rho 6G@1 for nitenpyram was as low as 0.48 nM and 3 nM, respectively. A multifunctional Zirconium-based NMOF (FMOF) was presented with 5,10,15,20-tetrakis(4-carboxyphenyl)porphyrin (H_2_TCPP) as an organic ligand for the determination of nitenpyram [66]. The PET between H_2_TCPP and nitenpyram caused an intensive quenching effect of FMOF. What is interesting is that FMOF can serve as a fluorescence sensor and photocatalyst to improve pesticides’ degradation (Figure 3d). The LOD of FMOF for nitenpyram was 0.03 µg/mL. MOF-5 [Zn_4_O(BDC)_3_DEF (BDC = 1,4-benzenedicarboxylate, DEF = diethylformamide)] was designed by Kumar et al. [67] for sensing a nitro group containing OPs including parathion, methyl parathion, paraoxon, and fenitrothion. MOF-5 exhibited strong green emission under the UV-light and, upon the addition of Ops, the fluorescence emission would be outstandingly quenched via the PET. The LOD for the four above OPs was 5 ppb. A Zirconium-based MOF (Zr-LMOF) with 1,2,4,5-tetrakis(4-carboxyphenyl) benzene (TCPB^4-^) as an organic ligand was used for the determination of parathion-methyl [68]. The rod-like Zr-LMOF showed robust blue fluorescence originating from the TCPB^4−^ ligand under UV-light. Owing to the PET, the fluorescence emission was significantly decreased when parathion-methyl was added. What is exciting, not only in aqueous solution but also in fresh vegetable samples, is that Zr-LMOF accomplished rapid, selective, and in situ detection of parathion-methyl with an LOD of 0.438 nM. With the similar strategy, a zinc-based NMOF probe with H_4_TCPB and tetrakis(4-carboxyphenyl)porphyrin (TCPP) as an organic ligand was reported for selective detection of parathion [69]. This probe emitted violet fluorescence originating from H_4_TCPB that can be selectively quenched by parathion through the PET with an LOD of 1.95 μg/L.

Recently, a series of Cadmium-based MOFs was synthesized with carboxylic acid ligands and the anthracene nitrogen-containing organic ligand [9,10-bis(N-benzimidazolyl)-anthracene] for serving as sensors [70], and one of these MOFs with bromo-terephthalic acid as the carboxylic acid ligand presented great potential to sensitively detect matrine. Upon the addition of matrine, the emission of the MOF derived from the organic ligand was almost completely quenched through the IFE and the FRET. Furthermore, the MOF can also efficiently detect picric acid and ferric ions through the quenching effect. The LOD for matrine was 30 ppb.

Wei et al. [71] fabricated a series of nanoscale eosin Y (EY)—embedded Zirconium-based MOFs (EY@DUT-52) with dual-emissive properties. Among these EY@ DUT-52, two (E@D1 and E@D3) exhibited great potential to sensitive detection of nitenpyram (a nicotine pesticides) in ethanol with a special residual fluorescence ratio measurement. EY@DUT-52 showed blue emission and orange emission originating from the NMOFs and EY embedded into DUT-52, respectively. E@D3 loaded more EY molecules than E@D1, which caused the difference in the relativity intensity of their dual-emission due to the FRET between EY and DUT-52. In the presence of nitenpyram, the resonant energy transfer from DUT-52 of E@D1 and E@D3 to nitenpyram happened, which resulted in the decreasing of blue emission and the quenching of orange emission because of the FRET between EY and DUT-52 being blocked (Figure 3e). Using the residual fluorescence ratio—the ratio of relative fluorescence intensity without nitenpyram and with nitenpyram as output signal—the LOD of E@D1 and E@D3 were 0.94 µM and 1.18 µM, respectively. 

Yu et al. [72] developed a 2D tetra-pyridyl calix[4]arene-decorated MOF nanosheet (MOF-Calix) for selectively sensing the glyphosate, a representative OP. Calix[4]arene on the surface of this NMOF possessed a highly accessible active site. In the presence of glyphosate, Calix[4]arene would selectively immobilize the target via a robust hydrogen bond, which can improve the rigidity of MOF-Calix and electron transfer. Meanwhile, the process of nonradiative decay was restrained while radiative decay was facilitated. Thus, the fluorescence emission of MOF-Calix increased to around 2.4-fold of its original emission within 1 min (Figure 3f). The LOD of MOF-Calix was as low as 2.25 µM.

#### 3.1.4. Mycotoxins

Mycotoxins, as secondary metabolites, the production of kinds of fungi like Aspergillus, Paecilomyces, and Fusarium shows a strong toxicity and poses a health risk to human beings, and rapid detection of mycotoxins is meaningful. In addition, each year, more than 2% agricultural products are contaminated by Mycotoxins, which almost lose all edible and commercial value [73]. NMOFs have attracted great concerns for fluorescence detection of mycotoxins because of their real-time, rapid, and relatively accurate detection.

Aflatoxins are highly toxic and carcinogenic secondary metabolite produced predominantly by *A. flavus* and *A. parasiticus*. Particularly, aflatoxin B_1_ (AFB_1_) showed the strongest harm to the livers of people and animals and was categorized as a carcinogen by the international cancer organization in 1988. Hu et al. [74] firstly accomplished the application of luminescent metal-organic frameworks for rapid and sensitive detection of AFB_1_. The designed MOF fluorescence sensor, Zn_2_(bpdc)_2_(tppe) (LMOF-241), exhibited robust blue-green fluorescence emission originated from the ligand 1,1,2,2-tetrakis(4-(pyridin-4-yl)phenyl)ethane(tppe) under the UV-light. Upon the addition of AFB_1_, the emission intensity was dramatically quenched. The quenching effect might be attributed to the PET from LMOF-241 to AFB_1_. The LOD of LMOF-241 for AFB_1_ was 46 ppb, which was much lower than the food and drug administration (FDA)-permitted maximum tolerant level (300 ppb). With the similar strategy, Zirconium-based NMOFs (Zr-CAU-24) with H_4_TCPB as an organic ligand was also used for sensitively sensing for AFB_1_ in aqueous solution and food sample [75]. Zr-CAU-24 exhibited robust blue fluorescence emission under the UV-light, which can be quenched by the presence of AFB_1_ within five minutes with high quenching efficiency (138,461 M^−1^), which is ascribed to the greatly strong orbital overlap between this NMOF and AFB_1_. In addition, high quenching efficiency for AFB_1_ was originated from the periodic structure of Zr-CAU-24 that outstandingly improved the electron transfer and endowed Zr-CAU-24 with great selective detection ability. The LOD to AFB_1_ can be up to 64 nM.

**Figure 4:**
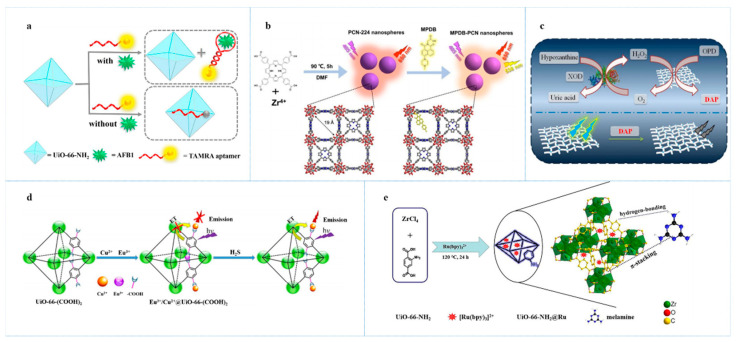
(**a**) Schematic illustration of the fluorescent aptasensor for AFB_1_ detection base on UiO-66-NH_2_, reprinted with modifications from Jia et al. [76]; (**b**) schematic representation of solvothermal synthesis of PCN-224 nanospheres and post-synthetic modification fabrication process of MPDB-PCN nanospheres, reprinted with modifications from Guo et al. [77]; (**c**) schematic illustration for the principle of the biosensor based on NH_2_-Cu-MOF nanosheet, reprinted with modifications from Hu et al. [80]; (**d**) schematic illustration of the fluorescence detection mechanism based on Eu^3+^/Cu^2+^@UiO-66-(COOH)_2_, reprinted with modifications from Zhang et al. [82]; (**e**) schematic illustration of the preparation process and the ratiometric fluorescence detection of melamine based on UiO-66-NH_2_@Ru Probe, reprinted with modifications from Lin et al. [89].

Jia et al. [76] developed a fluorescence sensing platform based on NMOF (UiO-66-NH_2_) and a TAMRA labeled-aptamer for effectively sensing AFB_1_ in milk, corn, and rice. The aptamer of this probe was immobilized on the surface of the NMOFs through van der Waals force. TAMRA, a fluorescence dye, was labeled on the aptamer, which can be extremely quenched through the charge transfer from TAMRA to the metal ion of NMOF. When AFB_1_ was present in the sample solution, the analytes would bind to the TAMRA aptamer and trigger the structure changing of the aptamer to form an internal loop accompanying the detaching from NMOF surface, which can cause fluorescence recovery (Figure 4a). This fluorescence probe exhibited a selective light-up response toward AFB_1_ with an LOD of 0.35 ng/mL.

3-nitropropionic acid (3-NPA) is a toxic neurotoxin that is mainly produced by moldy sugarcane and can potentially induce carcinogenicity, teratogenicity, mutagenicity, etc. Because sugarcane is a major raw source of sucrose, the facile detection of 3-NPA is meaningful. Gao et al. [77] fabricated a nanoscale ratiometric fluorescence sensor (MPDB-PCN) for determining 3-NPA in sugarcane juice via PH responding. MPDB-PCN was prepared by modification of PCN-224 with naphthalimide derivative (MPDB) and exhibited robust PH-depending fluorescence emission at 538 nm and 650 nm under the excitation at 405 nm, which were derived from MPDB and PCN-224, respectively. Upon the addition of 3-NPA, the intensity of fluorescence emission at 538 nm was gradually enhanced owing to the PET in MPDB while the emission at 650 nm was reduced due to the protonation of the ligand in PCN-224 (Figure 4b). With I_538_/I_650_ as ratiometric output, the LOD for 3-NPA was 15 µM. PH-responsive Zinc-based MOFs, [Zn_2_(tcpbp)(4,4′-bipy)_2_]·2DMA·6H_2_O were also used for highly sensitive detection of 3-NPA [78]. The sensing mechanism is a proton-induced quenching effect. In the presence of 3-NPA, the fluorescence emission of the MOFs was outstandingly quenched in the short time interval (3−4 s) through the protonation of organic ligand (H_4_tcpbp), which can be obviously observed by the naked eyes. By the means of neutralizing with NaOH, the fluorescence of the MOFs can be perfectly recovered. Owing to a sudden fluorescence transition of the MOFs in a narrow PH range, this MOF fluorescence sensor showed ultrahigh sensitivity to 3-NPA with an LOD as low as 1.0 µM.

#### 3.1.5. Spoilage Indicators

Food spoilage exists widely in daily life. The accident intake of spoiled food may lead to severe poisoning, various diseases, and even death [79]. During food spoilage, various substances are generated. Some of them are associated closely with the spoilage degree of food and considered as spoilage indicators. In recent years, the usage of NMOFs-based fluorescence sensors for sensing spoilage indicators has been explored.

Hypoxanthine (Hx) was often used as the spoilage indicator of fish. Hu et al. [80] presented an NMOF (NH_2_-Cu-MOF) for sensitively sensing Hx. In this case, NH_2_-Cu-MOF was used not only as an effective fluorescence sensor but also peroxidase mimic enzyme. Hx can react with oxygen and produce H_2_O_2_, then H_2_O_2_ would further yield hydroxyl radical via the catalysis of this NMOF. Next, o-phenylenediamine was added, which can react with the hydroxyl radical and produce the oxidized OPD (DAP) under the peroxidase mimic activity of NH_2_-Cu-MOF. DAP could outstandingly quench the fluorescence emission of NH_2_-Cu-MOF at 425 nm under the excitation at 338 nm (Figure 4c). In fish samples, Hx can also be determined by this sensing methods. The LOD for Hx was as low as 3.93 µM.

Zirconium-based MOF probes (Zr-BTDB-fcu-MOF) with 4,4’-(benzo[c] [1,2,5] thiadiazole-4,7-diyl) dibenzoic acid (H_2_BTDB) as an organic ligand were reported for turn-on determining methylamine (MA), a production generated during the spoilage of fish [81]. Upon the addition of MA, the fluorescence emission of this probe intensity was dramatically enhanced, which may be subjected to the hydrogen interaction between MA, and H_2_BTDB restricted the rotation of thiadiazole group on the H_2_BTDB and improved π conjugation. The LOD for MA was 66.2 nM.

Zhang et al. [82] designed an NMOF, Eu^3+^/Cu^2+^@UiO-66-(COOH)_2_, for ratiometric detection of hydrogen sulfide, a common spoilage indicator used for meat and fish. Eu^3+^/Cu^2+^@UiO-66-(COOH)_2_ showed characteristic fluorescence emission of Eu^3+^ at 615 nm and the emission at 393 nm derived from the H_4_btec ligands. Through the antenna effect, the emission derived from Eu^3+^ can be strongly enhanced while the emission derived from the ligand decreased. However, the existence of Cu^2+^ in this NMOF restricted this progress because Cu^2+^ is inclined to gain electrons. In addition, in the presence hydrogen sulfide, the restriction of antenna effect would be relieved because hydrogen sulfide possesses high affinity for Cu^2+^. With the I_615_/I_393_ as an output signal, the LOD of Eu^3+^/Cu^2+^@UiO-66-(COOH)_2_ for hydrogen sulfide was as low as 5.45 µM.

An aluminum-based MOF with 5-vinyl isophthalic acid (H_2_IPA-V) and 1,3-benzenedicaroxylic acid (H_2_IPA) as an organic ligand were also designed for fluorescence sensing of hydrogen sulfide [83]. By generating a ground state complex between the vinyl of organic ligand and hydrogen sulfide, a hydrogen sulfide was able to quench the fluorescence emission of this MOF within 10 s. This sensor can effectively determine hydrogen sulfide among various organic molecules and anions with the LOD value of 1.65 µM for hydrogen sulfide. Rarely, this fluorescence sensor can also selectively detect Pd^2+^ with an LOD of 110 nM.

#### 3.1.6. Illegal Additives

Illegal additives is one of the most severe problems in the field of food safety, which may have great threats to public health. Many groups have tried to detect illegal additives with NMOFs-based sensors.

Malachite green (MG) was forbidden to be used in aquaculture industry in China. However, the illegal usage of MG in fish farming still exists because MG possesses excellent antifungal and antibacterial ability as well as a low price [84]. Han et al. [85] presented an MOF sensor (Eu-TDA), Eu_2_(TDA)_4_(OOCCH_3_)_2_(H_2_O)_2_ (TDA = 2,5-thiophenedicarboxylic acid group, OOCCH_3_ = glacial acetic acid group), for sensitive determination of MG. Eu-TDA exhibited typical red fluorescence emission of Eu^3+^ under the UV-light. Upon the addition of MG, the emission remarkably decreased, which might be attributed to the IFE and the energy transfer from the MOF to MG. The LOD of Eu-TDA for MG was 0.0221 µM.

Clenbuterol is a common asthma drug in the clinical treatment. However, in animal husbandry, clenbuterol is illegally fed to livestock with a high dosage for improving muscle growth and reducing fat rate, which can pose a severe healthy risk to consumers [86]. UiO-66 was used for sensitive detection of clenbuterol in a urine sample of livestock as well as in aqueous solution [87]. The sensing mechanism is that the interaction between UiO-66 and clenbuterol in the ground state generates non-fluorescence complex. Therefore, upon the addition of clenbuterol, the fluorescence emission of UiO-66 was dramatically quenched via static quenching. The LOD of UiO-66 for clenbuterol was as low as 0.17 µM.

Melamine has a high percent of nitrogen and low price, and it was illegally added into infant formula and pet foods in recent years to increase apparent protein content, which can lead to severe damage to kidneys [88]. Recently, a fluorescent sensor (UiO-66-NH_2_@Ru) was synthesized by embedding tris(2,2bipyridyl)ruthenium chloride hexahydrate ([Ru(bpy)_3_]^2+^) into a nanoscale Zirconium-based MOF (UiO-66-NH_2_) [89], which accomplished sensitive detection of melamine in infant formula milk as well as aqueous solution. Dual-emission fluorescence sensors can more accurately sense target analytes compard to single emission fluorescence sensors because it can overcome the interference from solution environment, sensors concentration, instruments, and so on [90]. As dual-emission fluorescence sensors, UiO-66-NH_2_ emitted at 445 nm and 595 nm under the excitation wavelength of 350 nm. The two emission were derived from TPA-NH_2_ in UiO-66-NH_2_ and [Ru(bpy)_3_]^2+^, respectively. Owing to the FRET between TPA-NH_2_ and [Ru(bpy)_3_]^2+^, the fluorescence emission of the TPA-NH_2_ much decreased while the emission of [Ru(bpy)_3_]^2+^ was increased. In the presence of melamine, the robust interaction between melamine and TPA-NH_2_ via π-stacking, hydrogen bonding as well as donor–acceptor interaction outstandingly hindered the FRET progress, which caused the emission from TPA-NH_2_ to be greatly improved while the emission from [Ru(bpy)_3_]^2+^ slightly decreased (Figure 4e). With I_445_/I_595_ as the output signal, the LOD of UiO-66-NH_2_@Ru for melamine was 90 nM.

### 3.2. Ions

#### 3.2.1. Cations

Cations exist extensively in nature. Certain cations play important roles in life process [91]. However, some cations, such as heavy metal cations, may pose a significant risk to human health with high or low concentrations [92]. Thus, it is essential to accurately detect cations in food for securing public health. Over the past few years, lots of research groups tried to rapidly detect kinds of cations by the usage of NMOFs-based fluorescence sensors and have borne fruit [4]. In addition, many of them shared a similar sensing mechanism, in which NMOFs-based fluorescence sensors were selectively quenched by certain metal cations through the PET [93,94,95,96]. In this part, the latest and novel NMOFs sensors for cation detection will be introduced.

**Figure 5:**
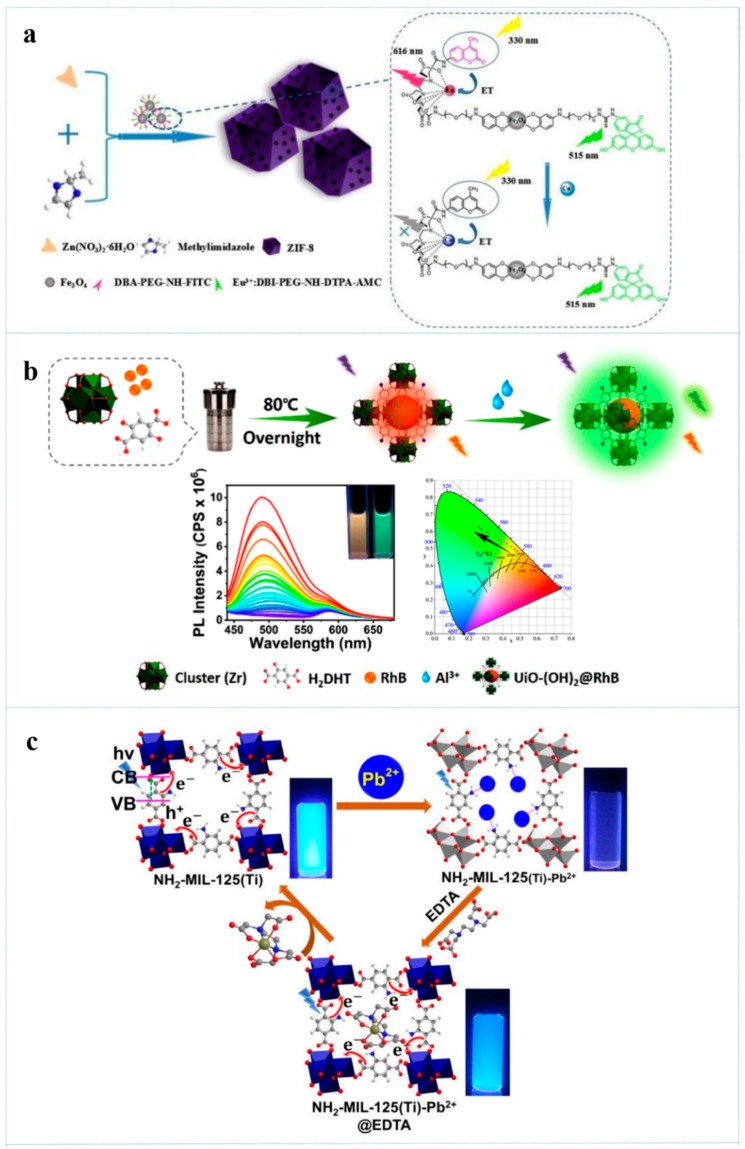
(**a**) Schematic illustration of the process for encapsulating the lanthanide complex modified Fe_3_O_4_ into ZIF-8 and concept for sensing Cu^2+^, reprinted with modifications from Wang et al. [99]; (**b**) schematic illustration of the fabrication of UiO-(OH)_2_@RhB and the sensing process to Al^3+^, reprinted with modifications from Zheng et al. [110]; (**c**) schematic of the fluorescence quenching and recovery mechanisms of NH_2_-MIL-125, reprinted with modifications from Venkateswarlu et al. [113].

The bio-accumulation of copper ion in organism can lead to some serious diseases, such as neurodegenerative diseases [97]. Yang et al. [98] prepared a Eu-based MOF, which showed intense red emission originated from Eu^3+^ through antenna effect. And the presence of Cu^2+^ inhibited the energy transfer from organic ligand to the center of Eu^3+^, resulting in strong quenching effect. While other ions exhibited negligible effect. Efficient determination of Cu^2+^ was accomplished by this strategy. Dual-emission fluorescent NMOFs-based probes were established by encapsulating isothiocyanate (FITC), a fluorescein, and Eu complex-functionalized Fe_3_O_4_ nanoparticles into ZIF-8 for ratiometric sensing Cu^2+^ [99]. These probes showed two emission peaks due to the emission of FITC and Eu complex at 515 nm and 616 nm, respectively. In the presence of Cu^2+^, Eu^3+^ in Eu complex groups can be replaced by Cu^2+^ because DTPA-Cu has a higher binding constant than DTPA-Eu, which will lead to quenching of emission at 616 nm. In this process, ZIF-8 served as a protective structure that can greatly improve sensitivity and selectivity by methods of not only preventing nanoparticles from aggregating but also amplifying signal (Figure 5a). With the FI_616_/FI_515_ as a ratiometric signal output, the LODs of this NMOFs-based probe for Cu^2+^ was 0.1 nM, which is 2 × 10^4^ times lower than the maximum allowable limit in drinking water.

Aluminum, as the most abundant metal element in earth [100], plays an essential role in our daily life. However, aluminum is also one of the hazardous metals [101] and excessive intake of it may result in various illnesses such as Alzheimer’s disease [102]. UiO-(OH)_2_@RhB was prepared for ratiometric determination of Al^3+^ by encapsulating red-emitting RhB into Zirconium-based NMOFs (UiO-(OH)_2_) with 2,5-dihydroxyterephthalic acid (H_2_DHT) as the organic ligand [103]. UiO-(OH)_2_@RhB emitted fluorescence both at 500 nm and 583 nm, which were originated from H_2_DHT and RhB, respectively. Upon the addition of Al^3+^, the emission of H_2_DHT dramatically enhanced while the emission of RhB almost remained unchanged. This progress was accompanied with the visible color change from orange to green within two minutes. The sensing mechanism was that H_2_DHT underwent ESIPT progress under UV-light and generated intramolecular hydrogen bonds, which weakened its fluorescence emission. In addition, the coordination between Al^3+^ and UiO-(OH)_2_@RhB can destroy the intramolecular hydrogen bond and resulted in dramatical enhancement of fluorescence emission (Figure 5b). With I_500_/I_583_ as a ratiometric signal readout, the LOD value of UiO-(OH)_2_@RhB was as low as 10 nM.

Cadmium ion is one of heavy metal ions with extreme toxicity and intake of it could induce cancer [104]. NMOFs probes (UiO-66-N=CH_2_) were synthesized for highly selective detection of Cd^2+^ with an LOD of 37.8 ppb [105]. UiO-66-N=CH_2_ was prepared through post-synthetic modification of UiO-66-NH_2_ by means of aldehyde-amine condensation reaction. In addition, these NMOF probes showed great thermal stability, pH-independence, and excellent luminescence stability in water. Upon the addition of Cd^2+^, the fluorescence emission of UiO-66-N=CH_2_ could be remarkably enhanced under excitation wavelength of 342 nm; this is because the coordination between Cd^2+^ and imine group in the ligand inhibits non-radiative transition and leads to a ligand-centered charge transfer.

Ferrum, as an important transition metal element, acts a crucial part in the biological process. Either too few or excessive intake of Fe^3+^ could result in different kinds of disorders as well as severe diseases like Parkinson’s disease and inflammation [106]. Yu et al. [107] constructed stable MOFs-based sensors (Eu-MOF and Tb-MOF) for Fe^3+^ by selecting rigid organic ligands of MOFs. Eu-MOF and Tb-MOF displayed strong water stability, heat resistance and luminescence stability at a wide PH range. Upon the addition of Fe^3+^, the characteristic fluorescence emission of sensors was almost completely quenched, which may be ascribed to the energy competition between analyte and sensors. Moreover, prepared sensors also showed outstanding performance for detecting CrO_4_^2−^ and Cr_2_O_7_^2−^. 2-(2-carboxyphenoxy)terephthalic acid (H_3_cpta) was first used for building MOFs by Gu et al. [108]. One of the prepared MOFs with Cd as metal node showed great potential for sensing Fe^3+^. This Cd-based MOF displayed robust emission derived from H_3_cpta, and Fe^3+^ can lead to essentially complete quenching effect through energy transfer from H_3_cpta to Fe^3+^. The LOD for Fe^3+^ was 0.21 mM. Amit Kumar et al. [109] reported an amine functionalized NMOF (IRMOF-3) for effectively Fe^3+^ detection. IRMOF-3 exhibited bluish fluorescence at 460 nm under UV-light, and, with the addition of Fe^3+^, the fluorescence quenching effect was clearly observed. The sensing mechanism is that the PET occurs with an amine group of IRMOF-3 as an electron donor and Fe^3+^ as an electron acceptor as well as surface complexes form. Owing to the outstanding advantages of the low price of IRMOF-3, this material possesses great potential in industrial production. The LOD of IRMOF-3 for Fe^3+^ in aqueous solution was 4.2 nM.

Mercury is known as one of the most toxic heavy metals, which could cause severe harm to people. Recently, novel NMOF probes were presented for selectively sensing of Hg^2+^ basing on fluorescein amidite (FAM)-labeled ssDNA(5′-56-FAM-ATT TGT TTT GTT TCC CCT TTC TTC TTT TCT TTT-3′) and NH_2_-MIL-101(Fe)@Fe_3_O_4_ [110]. FAM-ssDNA exhibits a strong fluorescence emission under UV-light. In addition, NH_2_-MIL-101(Fe)@Fe_3_O_4_, as a fluorescence quencher, can partly quench the fluorescence of FAM-ssDNA (to 65% of its initial fluorescence intensity) when FAM-ssDNA are effectively and rapidly adsorbed onto the surface of NH_2_-MIL-101(Fe)@Fe_3_O_4_ via electrostatic interaction and π–π stacking. The presence of Hg^2+^ would cause FAM-ssDNA to become duplex dsDNA via T-Hg-T interaction and resulted in further quenching (to 52% of its initial fluorescence intensity), which might be ascribed to the interaction between dsDNA and the Fe^3+^ on the NH_2_-MIL-101(Fe)@Fe_3_O_4_. The LOD of this probe for Hg^2+^ was as low as 8 nM.

A NMOF-based platform (Fe-MOF-NPs) was also reported for the determination of Hg^2+^ [111]. Fe-MOF-NPs were emissive in aqueous solution and the addition of Hg^2+^ can greatly enhance the emission of Fe-MOF-NPs accompanying with red shift, which can be attributed to the formation of coordination bond and soft-soft interactions between Fe-MOF-NPs and Hg^2+^. The LOD with Fe-MOF-NPs as a spectrofluorometric sensor was as low as 1.17 nM. Fe-MOF-NPs as a colorimetric sensor can also detect Hg^2+^ with even lower LOD. Meanwhile, Hg^2+^ was able to be detected by the naked eyes with the color of Fe-MOF-NPs solution changing from brown to yellow–green after adding Hg^2+^.

In recent years, lead poisoning that derived from contaminated food frequently occurred [112]. Detection of Pb^2+^ in food and drinking water is of vital importance in our daily life. A Titanium-based NMOF (NH_2_-MIL-125) with 2-aminoterephthalic acid (NH_2_-H_2_BDC) as an organic ligand was recently reported for the ultra-sensitive detection of Pb^2+^ [113]. NH_2_-MIL-125 emits fluorescence derived from NH_2_-H_2_BDC with a high photoluminescence quantum yield through ligand-to-cluster charge transfer (LCCT). In the presence of Pb^2+^, the florescence emission was strongly quenched due to the LCCT interaction between Pb^2+^ and amine group of NH_2_-H_2_BDC. Furthermore, the fluorescence of NH_2_-MIL-125 that was quenched by Pb^2+^ could be much recovered by EDTA on the account of the transfer of Pb^2+^ from NH_2_-MIL-125 to EDTA. In addition, the recovery effect of EDTA can reach more than 80% ever after five cycles (Figure 5c). The LOD for Pb^2+^ was as low as 7.7 pM in aqueous solution.

#### 3.2.2. Anions

Anion is extensively applied in kinds of fields like chemical, biological, and environmental process. Howver, the intake of some anion with excessive concentration has an adverse impact on public health [114]. Some NMOFs-based sensing platforms have been successfully developed for easy and accurate detection of anions.

Fluorine ion salts are used as additives in drinking water and the intake of fluorine ion is recognized as a severe health risk even with a low concentration [115]. Yang et al. [116] designed a nanosphere dual-emission fluorescence sensor (Boric acid-functional Eu-MOF) with 5-bop as organic ligand and Eu^3+^ as metal node for sensing F^−^. 5-bop can be excited to triplet state under UV-light, which will sensitize Eu^3+^ for strong red emission via the antenna effect. However, boric acid exhibits the antenna effect and thus dual-emission from Eu^3+^ (at 625 nm) and 5-bop (at 366 nm) was observed. Upon the addition of F^−^, the strong covalent interaction between the boric acid group and F^−^ recovered antenna effect, thus the emission at 625 nm gradually decreased and the emission at 366 nm obviously increased. The LOD with I_623_/I_366_ as a readout signal was as low as 2 µM. In addition, the color changing from red to blue with the addition of F^−^ can be observed by the naked eye, which provided a convenient detection method for F^−^.

Phosphate is related to many diseases. For example, people drinking water with high phosphate concentration in the long term may have a higher risk of kidney disease [117]. Novel single-component ratiometric fluorescence sensors, PCN-224, were designed for the quantitative determination of PO^4−^ [118]. PCN-224 was composed of ZrO clusters serving as PO^4−^ recognition sites and 5,10,15,20-tetrakis(4-methoxycarbonylphenyl) porphyrin (H_2_Tcpp) ligand serving as emission groups. Interestingly, under excitation wavelength of 380 nm, the ligand of PCN-224 possessed two different emission peaks at 440 nm and 650 nm, which are derived from the first excited singlet state S_1_ to the ground state S_0_ and the second excited singlet state S_2_ to S_0_, respectively. In the presence of PO^4−^, the fluorescence emission intensity of PCN-224 was enhanced while the emission intensity at 440 nm was not changed. With I_650_/I_440_ as the readout signal, the reported LOD for PO^4−^ reached 54 nM.

Chromate and dichromate are very soluble in water and carcinogenic [119]. Mukherjee et al. [120] prepared a series of NMOFs based on 5-azidoisophthalic acid (5N_3_-H_2_IPA), 4,4′- azopyridine (4,4-azp) and transition metal ions. One of them, [{Cd(5N_3_-IPA) (4,4′-azp)0.5(H_2_O)}(H_2_O)]_∞_, showed excellent performance in sensing CrO_4_^2−^ and Cr_2_O_7_^2−^. This NMOF emitted strong fluorescence at 435 nm under UV-light. In addition, in the presence of CrO_4_^2−^ or Cr_2_O_7_^2−^, the fluorescence was noticeably quenched due to the combined effect of energy transfer and competitive absorption. The process of fluorescence attenuation can be observed by the naked eyes. Meanwhile, fluorescence emission remained almost unchanged upon the addition of other anions. Based on this method, CrO_4_^2−^ and Cr_2_O_7_^2−^ can be sensitively detected with the LOD values of 11 nm and 4 nm, respectively.

### 3.3. Food-Borne Pathogen

Food-borne pathogen is easily spread in food and drinking water, posing a major threat to public health [121]. Considering the unique advantage of NMOFs in fluorescence detection, some NMOFs-based fluorescence sensors have been reported to sense a variety of food-borne bacteria. To date, the detection of food-borne pathogen through NMOFs-based fluorescence sensors was mainly accomplished by detecting bacteria themselves and their typical DNA or RNA sequences.

**Figure 6:**
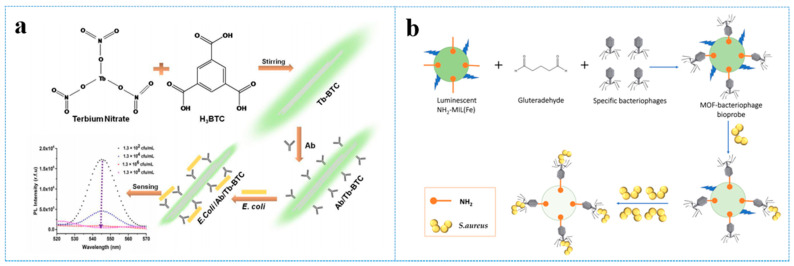
(**a**) Schematic diagram for the application of Ab/Tb-BTC for detecting *E. coli*, reprinted with modifications from Gupta et al. [123]; (**b**) schematic diagram for the application of NH_2_-MIL-53 (Fe) for sensing *S. aureus*, adapted from Bhardwaj et al. [124].

*Escherichia coli* (*E. coli*) is a Gram-negative, commensal microorganism usually found in the gut of warm-blooded animals. Some strains of *E. coli* can result in severe foodborne diseases, such as cholecystitis, bacteremia, traveler’s diarrhea, etc. [122]. Gupta et al. [123] presented rod-like fluorescence probes (Ab/Tb-BTC) based on Terbium MOFs (Tb-BTC, BTC = 1,3,5-benzenetricarboxylic acid) bio-interfaced with anti-*E. coli* antibodies, which accomplished highly selective, rapid and facile determination of *E. coli* in water and fruit juice. In this work, the anti-*E. coli* antibodies of the MOF can selectively bind to *E. coli* via antibody–antigen binding. With *E. coli* concentrations increasing, the strong fluorescence emission of these probes decreased in a short time, which can be subjected to the competitive absorption of excitation energy between Tb-BTC fluorophore and the antibody-antigen complex (Figure 6a). The LOD of Ab/Tb-BTC for *E. coli* reached 3 cfu/mL.

*Staphylococcus aureus* (*S. aureus*) contamination in food is usual and potentially harmful. Neha Bhardwaj et al. [124] connected bacteriophages with NMOFs, NH_2_-MIL-53 (Fe), via glutaraldehyde to sensitively and selectively detect *S. aureus* (Figure 6b). Upon the binding with *S. aureus*, the characteristic fluorescence emission of bacteriophage-NMOFs was quenched. The quenching mechanism was that NH_2_-MIL-53 (Fe) received lesser effective excitation after bacteriophage–bacteria interaction because of the competitive adsorption of excitation light between NH_2_-MIL-53 (Fe) and other components involved in this system. The LOD for *S. aureus* was 31 cfu/mL.

2D nanosheet MOFs-based fluorescence sensors (Cu-TCPP) were synthesized for selectively detecting three common food-borne pathogen genes, including invA gene of *Salmonella enterica*, prfA gene of *Listeria monocytogenes*, and toxR gene of *Vibrio parahemolyticus* [125]. Three ssDNA probes for invA gene, prfA gene, and toxR gene were labeled with fluorescence organic dyes—Texas red, Cy3, and FAM, respectively. Then, these probes were able to be efficiently absorbed onto the surface of Cu-TCPP, which can cause outstanding fluorescence quenching of the dye-labeled probes via the FRET. Upon the addition of specific pathogen genes, dsDNA formed accompanying with the combination of ssDNA probes and their complementary targets. Owing to weak affinity to Cu-TCPP, formed dsDNA detached from the MOF nanosheets and fluorescence recovered. The LOD for invA gene, prfA gene, and toxR gene were as low as 28 pM, 35 pM, and 15 pM, respectively.

The Ebolavirus transmitted as the result of handing or eating bushmeat such as bats, monkeys, and ape. Dysprosium-based MOFs were designed with N-carboxymethyl-(3,5-dicarboxyl) pyridinium bromide (H_3_CmdcpBr) as organic ligands, which served as a sensitive fluorescent sensor for the determination of Ebolavirus RNA sequences [126]. In this case, FAM-labeled ss-DNA that is complementary to the part of Ebolavirus RNA sequence served as the probe (P-DNA). In the presence of the MOFs, the interactions between MOFs and P-DNA caused the fluorescence intensity of P-DNA greatly decreased via hydrogen bonding, π–π stacking and electrostatic interactions. When target Ebolavirus RNA sequences were added, stable DNA/RNA hybrid duplexes formed and detached from the MOF because of weaker affinity, which resulted in fluorescence regeneration. The LOD for Ebolavirus RNA sequences was 160 pM. As a follow-up study, Copper-based MOFs, {[Cu(Cmdcp)(phen)(H_2_O)]_2_·9H_2_O}_n_ (H_3_CmdcpBr = N-carboxymethyl-(3,5-dicarboxyl)pyridinium bromide, phen = phenanthroline) were developed for synchronous determination of Ebolavirus conserved RNA sequences and Ebolavirus-encoded miRNA-like fragments through a similar mechanism with LODs of 60 pM and 206 pM, respectively [127].

## 4. Conclusions and Future Outlook

Encouraged by the excellent properties of NMOFs, a series of NMOFs fluorescence sensors for food safety have been developed in past decades as in Table 1. It would be easy to look at the challenges described in this Review and be concerned about the fate of NMOFs sensors. Although some achievements have been acquired, it is expected that NMOFs-based fluorescence sensors can play more and more important role in food safety aera. Food matrix contain various kinds of nutrition components and additives, which affect the pretreatment for food analysis. Remarkable progress needs to be made to improve the selectivity of sensors. Reasonable chemical modification or combining NMOFs with recognition groups like antibodies and aptamers could concentrate the target to improve the efficiency. Secondly, the related applications of NMOFs in food safety are still limited. A similarly thoughtful approach to NMOFs-based fluorescence sensors development should increase the likelihood that these potentially revolutionary tools can help to guarantee a safer and higher-quality food supply.

## Figures and Tables

**Table 1:** A summary of representative examples of NMOFs for food safety.

Analytes		Formula of MOF (Name)	LOD	Sample	Excitation/Emission	Linear Range	Size	References
Antibiotics	Tetracycline	not available (Dye@UiO-66@SiO2-Cit-Eu)	17.9 nM	Water; honey; milk	365 nm/617 nm and 430 nm	0.1 to 6 µM	around 130 nm	[39]
	Tetracycline	[In_2_(sbdc)_3_(H_2_O)_4_]∙(H_2_O)_8_ (In-sbdc)	0.28 µM	Water; milk; pork; fish	327 nm/377 nm	0 to 30 µM	around 250 nm	[40]
	Chlorotetracycline	[In_2_(sbdc)_3_(H_2_O)_4_]∙(H_2_O)_8_ (In-sbdc)	0.30 µM	Water; milk; pork; fish	327 nm/377 nm	0 to 30 µM	around 250 nm	[40]
	Oxytetracycline	[In_2_(sbdc)_3_(H_2_O)_4_]∙(H_2_O)_8_ (In-sbdc)	0.30 µM	Water; milk; pork; fish	327 nm/377 nm	0 to 30 µM	around 250 nm	[40]
	Doxycycline	not available (Al-MOF@Mo/Zn-MOF)	0.56 nM	Water; milk	330 nm/425 nm	0.001 to 46.67 µM	around 800 nm	[41]
	Tetracycline	not available (Al-MOF@Mo/Zn-MOF)	0.53 nM	Water; milk	330 nm/425 nm	0.001 to 46.67 µM	around 800 nm	[41]
	Oxytetracycline	not available (Al-MOF@Mo/Zn-MOF)	0.58 nM	Water; milk	330 nm/425 nm	0.001 to 46.67 µM	around 800 nm	[41]
	Chlortetracycline	not available (Al-MOF@Mo/Zn-MOF)	0.86 nM	Water; milk	330 nm/425 nm	0.001 to 46.67 µM	around 800 nm	[41]
	Doxycycline	not available (Eu-In-BTEC)	47 nM	Water; fish; urine	365 nm/526 nm and 617 nm	0 to 6 µM (526 nm)/0 to 3 µM (617 nm)	around 600 nm	[51]
	Ofloxacin	{[Zn_3_(OH)(bmipia)(H_2_O)_3_]_4_·[Zn(H_2_O)6.5]_2_}*_n_*(FCS-3)	0.52 µM	Water	401 nm/452 nm	0 to 0.0215 mM	not available	[43]
	Tetracycline	[DMA^+^]_2_[Tb_9_(μ_3_- OH)_8_(μ_2_-OH)_3_(H_2_O)_3_ (C_21_H_11_O_6_)_6_] ·11DMF·23H_2_O (Tb-L1)	8 ng/mL	Ethanol	290 nm/543 nm and 345 nm	0.06 to 10 μg/mL	not available	[44]
	Nitrofurazone	{[Cd_3_(TDCPB)·2DMAc]·DMAc·4H_2_O}*_n_* (complex 1)	not available	DMAc solution	318 nm/358 nm	not available	not available	[45]
	Nitrofurantoin	{[Cd_3_(TDCPB)·2DMAc]·DMAc·4H_2_O}*_n_* (complex 1)	not available	DMAc solution	318 nm/358 nm	not available	not available	[45]
	Nitrofurazone	not available (RhB@ZIF-8)	0.26 µM	Water	360 nm/580 nm	0 to 0.12 mM	around 50 nm	[46]
	Nitrofurantoin	not available (RhB@ZIF-8)	0.47 µM	Water	360 nm/580 nm	0 to 0.12 mM	around 50 nm	[46]
	Tetracycline	not available (RhB@ZIF-8)	0.11 µM	Water	360 nm/580 nm	0 to 0.046 mM	around 50 nm	[46]
	Oxytetracycline	not available (RhB@ZIF-8)	0.14 µM	Water	360 nm/580 nm	0 to 0.046 mM	around 50 nm	[46]
	Nitrofurazone	not available (FSS@ZIF-8)	0.31 µM	Water	380 nm/540 nm	0 to 0.038 mM	around 50 nm	[46]
	Nitrofurantoin	not available (FSS@ZIF-8)	0.35 µM	Water	380 nm/540 nm	0 to 0.12 mM	around 50 nm	[46]
	Tetracycline	not available (FSS@ZIF-8)	0.17 µM	Water	380 nm/540 nm	not available	around 50 nm	[46]
	Oxytetracycline	not available (FSS@ZIF-8)	0.16 µM	Water	380 nm/540 nm	not available	around 50 nm	[46]
	Nitrofurazone	[Me_2_NH_2_][Tb_3_(dcpcpt)_3_(HCOO)]∙DMF∙15H_2_O (Tb-dcpcpt)	0.502 µM	Water	300–390 nm/545 nm	0 to 0.1 mM	not available	[47]
	Nitrofurantoin	[Me_2_NH_2_][Tb_3_(dcpcpt)_3_(HCOO)]∙DMF∙15H_2_O (Tb-dcpcpt)	0.448 µM	Water	300–390 nm/545 nm	0 to 0.1 mM	not available	[47]
	Ciprofloxacin	[Me_2_NH_2_][Tb_3_(dcpcpt)_3_(HCOO)]∙DMF∙15H_2_O (Tb-dcpcpt)	0.21 µM	Water	300–390 nm/441 nm and 583 nm	0 to 0.1 mM	not available	[47]
	Norfloxacin	[Me_2_NH_2_][Tb_3_(dcpcpt)_3_(HCOO)]∙DMF∙15H_2_O (Tb-dcpcpt)	0.17 µM	Water	300–390 nm/441 nm and 583 nm	0 to 0.1 mM	not available	[47]
	Ceftriaxone sodium	not available (Cd-MOF)	55 ppb	Water	260 nm/288 nm	not available	not available	[48]
	Chloramphenicol	not available [Zn•(BA)•(BBI)]	12 ppb	Water; serum samples	270 nm/290 nm	0 to 5 × 10^−5^ mM	around 500 nm	[49]
	Ceftriaxone	not available [Zn•(BA)•(BBI)]	3.9 ppb	Water; serum samples	270 nm/290 nm	0 to 5 × 10^−5^ mM	around 500 nm	[49]
	Ascorbic acid	not available [Zn•(BA)•(BBI)]	1.6 ppb	Water; serum samples	270 nm/290 nm	0 to 5 × 10^−5^ mM	around 500 nm	[49]
	Patulin	Zn(TA)·(H_2_O).(DMF) (ZnMOF)	0.06 µM	Water; apple juice	315 nm/425 nm	0.1 to 10 μM	around 500 nm	[50]
Food additives	Nitrite	{[Tb(CA)(OA)_0.5_(H_2_O)_2_]·H_2_O}*_n_*(Tb-MOF)	28.25 nM	Water	295 nm/544 nm	0 to 15.6 µM	not available	[54]
	Formaldehyde	not available (UiO-66-NH_2_)	4 ppm	Water	328 nm/440 nm	10 to 100 ppm	around 200 nm	[56]
	Formaldehyde	not available (Eu/Zr-MOF)	0.2 ppm	Water	365 nm/465 nm and 615 nm	0 to 160 ppm	around 50 nm	[57]
	Tertiary butylhydroquinone	[Sm (DCPP)(H_2_O)_4_]*_n_*·2nH_2_O (Sm-MOF)	5.6 ng/mL	Water; soybean oil	300 nm/643 nm	0 to 120 µg/mL	not available	[59]
	Sesamol	[Sr(BDC)·DMAC·H_2_O]*_n_* (Sr-MOF)	4.2 µM	Ethanol	294 nm/545 nm and 330 nm	10^−7^ to 8 × 10^−4^ M	not available	[61]
Pesticides	Parathion-methyl	not available (ZnPO-MOF)	0.456 nM	Water	365 nm/420 nm	1.0 µg/kg to 10 mg/kg	around 130 nm	[64]
	Nitenpyram	[Cd_2_(tib)(btb)(H_2_O)_2_]∙NO_3_∙2DMF (1)	0.48 nM	DMF; water	340 nm/370 nm and 600 nm	0 to 1.1 nM	not available	[65]
	Nitenpyram	[Cd_2_(tib)(btb)(H_2_O)_2_]∙NO_3_∙2DMF (1)	3 nM	DMF; water	340 nm/370 nm and 600 nm	0 to 0.2 nM	not available	[65]
	Nitenpyram	not available (FMOF)	0.03 µg/mL	Water; soil	415 nm/650 nm	0.05 to 10 µg/mL	around 90 nm	[66]
	Parathion	Zn_4_O(BDC)_3_DEF (MOF-5)	5 ppb	Water	330 nm/493 nm	5 to 600 ppb	around100 nm	[67]
	Methyl parathion	Zn_4_O(BDC)_3_DEF (MOF-5)	5 ppb	Water	330 nm/493 nm	5 to 600 ppb	around100 nm	[67]
	Paraoxon	Zn_4_O(BDC)_3_DEF (MOF-5)	5 ppb	Water	330 nm/493 nm	5 to 600 ppb	around100 nm	[67]
	Fenitrothion	Zn_4_O(BDC)_3_DEF (MOF-5)	5 ppb	Water	330 nm/493 nm	5 to 600 ppb	around100 nm	[67]
	Parathion-methyl	not available (Zr-LMOF)	0.438 nM	Water; Lettuce; Cowpea	365 nm/420 nm	70 µg/kg to 5.0 mg/kg	around 900 nm	[68]
	Parathion	not available (Zn-MOF)	1.95 µg/L	Water	275 nm/380 nm	5 µg/L to 1 mg/L	around 500 nm	[69]
	Matrine	not available (F_4_)	30 ppb	DMF	324 nm/423 nm	0 to 5 ppm	not available	[70]
	Nitenpyram	not available (EY@ DUT-52)	0.94 µM	Ethanol	340 nm/380 nm and 555 nm	0 to 0.1 mM	around 600 nm	[71]
	Nitenpyram	not available (EY@ DUT-52)	1.18 µM	Ethanol	340 nm/380 nm and 567 nm	0 to 0.1 mM	around 600 nm	[71]
	Glyphosate	{[Cd_2_(5-NO_2_-BDC)_2_L(MeOH)]∙2MeOH}*_n_* (MOF-Calix)	2.25 µM	Water	281 nm/329 nm	2.5 to 45 µM	around 100 nm	[72]
Mycotoxins	Aflatoxin B1	Zn_2_(bpdc)_2_(tppe) (LMOF-241)	46 ppb	Water	340 nm/500 nm	not available	not available	[74]
	Aflatoxin B1	not available (Zr-CAU-24)	64 nM	Water; spiked walnut; almond beverages	340 nm/410 nm	0.075 to 25 µM	around 900 nm	[75]
	Aflatoxin B1	not available (UiO-66-NH_2_)	0.35 ng/mL	Water; corn; rice; milk	560 nm/580 nm	0 to 0.5 ng/mL and 1.5 to 3.0 ng/mL	around 500 nm	[76]
	3-nitropropionic acid	not available (MPDB-PCN)	15 µM	Sugarcane juice	405 nm/538 nm and 655 nm	0 to 800 μM	around 90 nm	[77]
	3-nitropropionic acid	[Zn_2_(tcpbp)(4,4′-bipy)_2_] (1)	1.0 µM	Colloidal solution	320 nm/393 nm	0 to 18 µM	not available	[78]
Spoilage indicators	Hypoxanthine	not available (NH_2_-Cu-MOF)	3.93 µM	Water; fish samples	338 nm/425 nm	10 to 2000 µM	around 700 nm	[80]
	Methylamine	not available (Zr-BTDB-fcu-MOF)	66.2 nM	Water	400 nm/515 nm	not available	not available	[81]
	Hydrogen Sulfide	Zr_6_O_4_(OH)_4_(O_2_C-C_6_H_2_-CO_2_-(CO_2_H)_2_)_6_·*x*H_2_O [UiO-66-(COOH)_2_]	5.45 µM	Water	305 nm/365 nm	not available	around 800 nm	[82]
	Hydrogen Sulfide	not available (CAU-10-V-H)	1.65 µM	HEPES buffer	365 nm/420 nm	70 µg/kg to 5.0 mg/kg	around 900 nm	[83]
Illegal additives	Malachite Green	Eu_2_(TDA)_4_(OOCCH_3_)_2_(H_2_O)_2_ (Eu-TDA)	0.0221 µM	Ethanol	302 nm/615 nm	not available	not available	[85]
	Clenbuterol	not available (UiO-66)	0.17 µM	Water; urine sample	290 nm/396 nm	4.0 to 40 ng/mL	not available	[87]
	Melamine	not available (UiO-66-NH_2_@Ru)	0.27 µM	Water; infant formula milk	350 nm/445 nm and 595 nm	0.27 to 110 µM	around 300 nm	[89]
Cations	Cu^2+^	[Eu_3_(bcpb)_4_(µ-HCOO)(µ-H_2_O)(H_2_O)_2_(DEF)]_n_	not available	DEF	338 nm/614 nm	0.05 to 2.5 mM	not available	[98]
	Cu^2+^	not available (Eu^3+^:AMC-DTPA-NH-PEG-DBI-Fe_3_O_4_-DBI-PEG-NH-FITC@ZIF-8)	0.1 nM	water	616 nm/515 nm	0.1 to 1 nM	around 100 nm	[99]
	Al^3+^	not available (UiO-(OH)_2_@RhB)	10 nM	Water; grain beans	420 nm/500 nm and 583 nm	0 to 10 μM	around 200 nm	[103]
	Cd^2+^	not available (UiO-(OH)_2_@RhB)	37.8 ppb	Water	342 nm/468 nm	0 to 500 µM	around 100 nm	[103]
	Fe^3+^	[Eu_2_(HICA)(BTEC)(H_2_O)_2_]_n_ (Eu-MOF)	not available	Water	300 nm/616 nm	0 to 50 µM	not available	[107]
	Fe^3+^	{[Tb_2_(HICA)-(BTEC)(H_2_O)_2_]·2.5H_2_O}_n_ (Tb-MOF)	not available	Water	310 nm/545 nm	0 to 40 µM	not available	[107]
	Fe^3+^	{[Cd_3_(μ_6_-cpta)_2_(py)_2_]·5H_2_O}_n_ (4)	0.21 mM	Water	375 nm/448 nm	10^-4^ to 10^-3^ M	not available	[108]
	Fe^3+^	not available (IRMOF-3)	4.2 nM	Water	360 nm/460 nm	0.1 to 4.0 µM	around 250 nm	[109]
	Hg^2+^	not available [NH_2_-MIL-101(Fe)@Fe_3_O_4_]	8 nM	Water	495 nm/520 nm	2 to 20 nM	around 150 nm	[110]
	Hg^2+^	not available [Fe(II)-MOF-NPs]	1.17 nM	Water	330 nm/422 nm	1.0 nM to 1.0 µM	around 200 nm	[111]
	Pb^2+^	not available [NH_2_-MIL-125(Ti)]	7.7 pM	Water	360 nm/450 nm	0 to 11 nM	around 500 nm	[113]
Anions	F^−^	not available (LMOFs)	2 µM	Water	275 nm/366 nm and 625 nm	4 to 80 µM	around 800 nm	[116]
	PO^4−^	not available (PCN-224)	54 nM	Water	380 nm/650 nm and 440 nm	0 to 10 µM	around 90 nm	[118]
	CrO_4_^2−^	[{Cd(5N_3_-IPA) (4,4′-azp)0.5(H_2_O)}(H_2_O)]_∞_(3)	11 nM	Water	350 nm/435 nm	not available	around 150 nm	[120]
	Cr_2_O_7_^2−^	[{Cd(5N_3_-IPA) (4,4′-azp)0.5(H_2_O)}(H_2_O)]_∞_(3)	4 nM	Water	350 nm/435 nm	not available	around 150 nm	[120]
Food-borne pathogen	*Escherichia coli*	not available (Tb-BTC)	3 cfu/mL	Water; fruit juice	292 nm/545 nm	1.3 × 10^2^ to 1.3 × 10^8^ cfu/mL	not available	[123]
	*Staphylococcus aureus*	not available [NH_2_-MIL-53(Fe)]	31 cfu/mL	Water	300 nm/430 nm	40 to 4 × 10^8^ cfu/mL	around 700 nm	[124]
	invA gene of *Salmonella enterica*	not available (Cu-TCPP)	28 pM	Water	589 nm/616 nm	0.5 to 15 nM	around 900 nm	[125]
	prfA gene of *Listeria monocytogenes*	not available (Cu-TCPP)	35 pM	Water	540 nm/562 nm	0.1 to 12 nM	around 900 nm	[125]
	toxR gene of *Vibrio parahemolyticus*	not available (Cu-TCPP)	15 pM	Water	490 nm/520 nm	0.1 to 9 nM	around 900 nm	[125]
	Ebolavirus RNA sequences	{[Dy(Cmdcp)(H_2_O)_3_](NO_3_)·2H_2_O}*_n_* (1)	160 pM	Water	not available	not available	not available	[126]
	Ebolavirus conserved RNA sequences	{[Cu(Cmdcp)(phen)(H_2_O)]_2_·9H_2_O}*_n_* (1)	60 pM	Water	492 nm/518 nm	0 to 60 nmol/L	not available	[127]
	Ebolavirus-encoded miRNA-like fragment	{[Cu(Cmdcp)(phen)(H_2_O)]_2_·9H_2_O}*_n_*(1)	206 pM	Water	578 nm/604 nm	0 to 60 nmol/L	not available	[127]

## Data Availability

We did not report any new data in this review.

## Abbreviations

MOFs	Metal-Organic Frameworks
NMOFs	Nanoscale MOFs
TC	Tetracycline
FRET	Fluorescence Resonance Energy Transfer
TET	Tetracycline
CTC	Chlorotetracycline
OTC	Oxytetracycline
DOX	Doxycycline
IFE	Inner-filter Effect
PET	Photo-induced Electron Transfer
RhB	Rhodamine B
FSS	Fluorescein disodium salt
NFAs	Nitrofurans
OFX	Ofloxacin
NFZ	Nitrofurazone
NFT	Nitrofurantoin
CPFX	Ciprofloxacin
NFX	Norfloxacin
CRO	Ceftriaxone
CHL	Chloramphenicol
TBHQ	Tertiary Butylhydroquinone
OPs	Organophosphate Pesticides
AFB1	Aflatoxin B1
3-NPA	3-nitropropionic Acid
Hx	Hypoxanthine
MA	Methylamine
MG	Malachite Green
E. coli	Escherichia coli
S. aureus	Staphylococcus aureus
